# PVDF-Based Piezo-Catalytic Membranes—A Net-Zero Emission Approach towards Textile Wastewater Purification

**DOI:** 10.3390/polym16050699

**Published:** 2024-03-04

**Authors:** Amna Siddique, Hifza Nawaz, Shumaila Razzaque, Anila Tabasum, Hugh Gong, Humaira Razzaq, Muhammad Umar

**Affiliations:** 1 Department of Chemistry, University of Wah, Quaid Avenue, Wah 47040, Pakistan; uw-20s-chm-phd-002@student.uow.edu.pk (A.S.); aneela.tabassum@uow.edu.pk (A.T.); 2 Department of Materials, University of Manchester, Oxford Road, Manchester M13 9PL, UK; hafizahifza.nawaz@manchester.ac.uk (H.N.); hugh.gong@manchester.ac.uk (H.G.); 3 Institute of Physical Chemistry, Polish Academy of Sciences, Kasprzaka44/52, 01-224 Warsaw, Poland; srazzaque@ichf.edu.pl

**Keywords:** PVDF, piezo-catalysis, piezoelectric materials, degradation, net-zero emissions, reactive oxygen species (ROS)

## Abstract

Among the various water purification techniques, advancements in membrane technology, with better fabrication and analysis, are receiving the most research attention. The piezo-catalytic degradation of water pollutants is an emerging area of research in water purification technology. This review article focuses on piezoelectric polyvinylidene difluoride (PVDF) polymer-based membranes and their nanocomposites for textile wastewater remediation. At the beginning of this article, the classification of piezoelectric materials is discussed. Among the various membrane-forming polymers, PVDF is a piezoelectric polymer discussed in detail due to its exceptional piezoelectric properties. Polyvinylidene difluoride can show excellent piezoelectric properties in the beta phase. Therefore, various methods of β-phase enhancement within the PVDF polymer and various factors that have a critical impact on its piezo-catalytic activity are briefly explained. This review article also highlights the major aspects of piezoelectric membranes in the context of dye degradation and a net-zero approach. The β-phase of the PVDF piezoelectric material generates an electron–hole pair through external vibrations. The possibility of piezo-catalytic dye degradation via mechanical vibrations and the subsequent capture of the resulting CO_2_ and H_2_ gases open up the possibility of achieving the net-zero goal.

## 1. Introduction

Today’s world faces many energy crises, which restrict the availability of fresh drinking water [1]. Wastewater treatment by conventional methods is becoming one of the greatest challenges around the world. Evolving environmental issues, the increasing global population, and urbanization have had significant effects on the quality and quantity of drinking water [2]. Various human activities, such as elevated industrialization and agricultural and domestic use, cause water pollution on a large scale [3]. The demand for dyestuffs in the textile sector is increasing with the passage of time and the fast growth of the fashion sector [4]. Most carcinogenic dye components from the textile industry are directly discharged into surface water without any treatment. These untreated dye effluents contain toxic and non-biodegradable compounds, which raise serious environmental concerns [5]. Although technological advancements have led to sustainable, environmentally friendly, and robust water purification techniques, water purification with energy-efficient methods is still a major challenge for scientists. Materials scientists have been extensively exploring the use of natural energy resources (solar and mechanical energy) in recent years [6]. Harvesting mechanical energy during the treatment of water-polluted resources has also introduced the concept of piezo-catalysis [7]. Piezo-catalysis uses mechanical energy to facilitate the electro-catalytic processes of pollutant molecules using mechano-electric materials. The electronic state of piezo-materials induces a potential difference, reacting with the attached pollutant molecules and converting them into small molecules (CO_2_ + H_2_O) [8]. The concept of piezoelectricity was introduced in 1880 by J.P. Curie. However, this phenomenon was explained via material structures in the 20th century. Piezoelectric properties can be effected by the chemical structure and also depend on the method of fabrication [9,10].

Different organic and inorganic piezoelectric materials have been explored because of their energy conservation applications. Inorganic materials such as ceramic, quartz, perovskites [10,11,12], and/or metal oxide nanoparticles [13] show good piezoelectric properties but have various technological limitations. The disadvantages of such inorganic materials include a poor molding capacity, limitations in size, high brittleness, and sometimes solubility in organic solvents. To overcome such limitations, these inorganic materials are doped in piezoelectric polymers. These polymers possess multiple properties and modifiable chemical structures. Various piezoelectric polymers in amorphous and semi-crystalline forms have great potential for future application. For example, polyvinylidene fluoride [14], polyacrylonitrile (PAN) [15], polytetrafluoroethylene (PTFE) [16], polyvinyl chloride (PVC), polyamide and polyimides (PI) [17], epoxy resins [18], polyolefins, polyvinyl acetate (PVA) [19,20], poly(methyl methacrylate) (PMMA), polyuria, polysulfone (PSF), and polycarbonates (PC) exhibit piezo-catalytic properties when combined with inorganic nanomaterials.

Polyvinylidene fluoride is a leading polymer in the field of piezoelectric materials due to its crystalline nature and relatively simple chemical nature in the beta form, where fluorine atoms are located on the same side of the chain as its permanent dipole, and it has a relatively high dielectric constant. Polyvinylidene fluoride (PVDF) [7] is the most utilized polymer in wastewater treatment membranes because of its excellent properties, such as its hydrophobic nature, antifouling properties, aging resistance, mechanical strength, high thermal resistance, and mechanical and chemical stability. Zheng et al. prepared a PVDF membrane with remarkable dye rejection. For textile wastewater treatment, PVDF-based membranes have been attracting significant attention [21]. PVDF is a material that accelerates the piezo-catalytic degradation (mechanical vibration accelerates the breakdown of organic molecules) of dyes and pollutants [9]. For the beta phase of PVDF, the piezo-catalytic activity can be increased by incorporating clay, ceramics, metal oxides, carbon black, graphene, carbon nanotubes, inorganic salts, carbon nonbars, and other polymers [14]. TD Raju et al. [5] reported the preparation of a PVDF matrix incorporated with zinc stannate (ZnSnO_3_) and cobalt (II,III) oxide (Co_3_O_4_) nanoparticles, which was used as a thermoplastic mold (PZC) for piezo-catalytic dye degradation. The ZnSnO_3_ and Co_3_O_4_ nanoparticles improved the piezo-catalytic activity of PVDF composites and showed 100% dye (RhB, MB, and RMB) degradation in 20 min. Table 1 shows the PVDF-based polymeric composite membranes used in previously reported works to remove different cationic and anionic dyes from textile materials.

B. Bagchia et al. [23] prepared novel multifunctional nanocomposites by combining piezoelectric molybdenum sulfide (MoS_2_) and a polyvinylidene fluoride (PVDF) polymer, which helped in the degradation of some carcinogenic dyes under a piezo-catalytic effect via ultrasound vibration within 20 minutes. Nanoflowers of MoS_2_-induced β-crystals in PVDF led to the self-poling of PVDF and increased its piezo-catalytic abilities [23]. F Orudzhev et al. [31] synthesized a new PVDF-based piezo-photocatalytic membrane via the incorporation of α-Fe_2_O_3_ nanoparticles, which resulted in an electroactive β-phase enhancement in PVDF and helped in the decomposition of methylene blue (MB) under the influence of ultrasonic vibration. α-Fe_2_O_3_ nanoparticles facilitate the electroactive β-phase of PVDF and help in dye degradation via the piezo-catalytic effect. G. Singh et al. [29] prepared flexible PVDF-doped lithium niobate (LiNbO_3_) composite films decorated with Ag NPs, which helped in cationic and anionic dye degradation under ultrasonication. β-phase PVDF can be obtained by solvent casting, phase transition, and the addition of nucleating fillers, which increase the piezo-catalytic activity of PVDF-based membrane materials [6].

Beta-phase PVDF materials show the piezoelectric phenomenon [8] through the alignment of the molecular dipole under the influence of a mechanical force. An electric field induced in piezoelectric materials generates free charges and helps in the micro-electrolysis of water for the production of reactive oxygen species (ROS), including ^•^OH, ^•^O^−^, ^•^HO_2_, and H_2_O_2_. These reacting molecules created by the mechanical activation of triboelectric materials can oxidize and degrade the harmful and carcinogenic pollutants that are discharged by the food, pharmaceutical, and textile industries [23].

This study explores piezoelectric materials with a specific focus on piezo-polymers such as polyvinylidene difluoride (PVDF). The investigation emphasizes PVDF’s piezo-catalytic enhancement, particularly in refining its crucial beta phase. This phase plays a significant role in achieving energy-efficient solutions, especially in water purification and membrane formation. The proposed method utilizes mechanical vibration, which eliminates the need for excessive energy consumption. This innovative approach not only filters water but also degrades pollutants, aligning with the goal of achieving net-zero pollution in water purification.

The objective of this review is to describe the role of PVDF-based piezoelectric membranes as passive, effective, cheap, and eco-friendly tools for wastewater treatment and the degradation of dyes. This review gives clear directions for the selection of an appropriate nanocomposite material for incorporation with PVDF to achieve its desired structural morphology and potential application as an enhanced piezoelectric material for net-zero water treatment. Meanwhile, this review also presents a comprehensive comparison and considers the mechanism of heterojunctions in piezoelectric materials. To the best of our knowledge, this is the first work to discuss how PVDF-based piezoelectric membranes can be used to achieve net-zero goals via minimal energy utilization. The selected works also reveal the current potential to achieve energy efficiency via hydrogen production and CO_2_ recycling to produce usable chemicals for future energy conservation.

## 2. Classification of Piezoelectric Materials

There are various types of piezo-catalytic materials, such as inorganic, organic, and polymeric piezoelectric materials.

### 2.1. Piezoelectric Biopolymers

In the 1950s, it was found that biomaterials (cellulose and collagen) exhibit polarization within their polymer chain structures and show piezoelectric properties [32]. Later on, various biomaterials were explored as piezoelectric materials, such as polylactic acid (PLA), amino acids [33], proteins, and polyhydroxy butyrate (PHB) [32,33,34,35]. These piezoelectric materials not only show piezoelectric properties but also have excellent conductive, physical, and optical properties. Because of these multiple properties, these materials are used in electronic devices, sensors, optical and other applications. Computational studies are being used for calculations in quantum mechanics, which helps to predict the piezoelectric properties of organic molecules [36], such as biomolecular crystals, small peptides, and amino acids. By tuning the molecular chemistry of biomolecules [37] by generating supramolecular dipoles, it may be possible to form renewable piezoelectric biomolecules. Synthetic biomaterials have shown better piezoelectric properties [38]. Among the various piezoelectric biomaterials, polyvinylidene difluoride has outstanding piezoelectricity due to its flexibility and beta-phase molecular structure. Applications of PVDF and its copolymers exist in areas such as bone growth, cell differentiation, muscle regeneration in tissue engineering, and energy harvesting systems [39]. The piezoelectricity of peptides [40] is strong due to their nanostructures and noncovalent interactions, such as van der Waals interactions, electrostatic interactions, and hydrophobic and stacking interactions, as well as hydrogen bonding, which is used for self-linkages in peptides [41]. The non-centrosymmetric crystal structures of some self-assembled nanostructures help them to attain excellent piezoelectric properties. Such biocompatible peptides have many applications in medical delivery systems [42], energy storage devices [43], cell cultures [44], ultrasensitive sensors, and power generation systems [43]. Crystals, which are based on biomolecules like proteins and amino acids, usually have poorer piezoelectric properties as compared to peptides [45].

The more favorable arrangement and smaller size of peptide structures facilitate enhanced piezoelectric properties [46] compared to larger molecules like proteins and amino acids. The chemical composition, growing process, and crystal structure can control the piezoelectric properties of piezoelectric biopolymers [47]. These biopolymers can be explored further for piezoelectric applications, and uniform polarization and strong piezoelectricity can be developed in these materials [32]. These piezoelectric biopolymers have great potential for application in biomaterial-based piezoelectric devices.

### 2.2. Inorganic Piezoelectric Materials

Many organic and inorganic materials can convert mechanical vibration into electric energy via their piezoelectric properties. Many inorganic materials, such as elongated (Ba,Sr) TiO_3_ nanowires, ZnO, and BaTiO_3_, which show piezo-catalysis, are non-centrosymmetric, which leads to electric dipoles in these materials [48,49,50]. The asymmetric nature of the atoms in the crystal lattice makes them sensitive to stress, stretching, or squeezing, which cause the displacement of the atoms from their original positions and cause positive and negative charges to appear on the outer faces of the crystals [51]. These inorganic materials also enhance the piezo-catalytic process, which helps to degrade organic pollutants, and they have various applications in the field of wastewater treatment. They may be used as additives in the polymeric matrices of piezoelectric materials. Table 2 illustrates the inorganic piezoelectric materials and their properties, applications, and necessary conditions for the degradation of organic pollutants.

These inorganic piezoelectric materials show some limitations and disadvantages. These limitations include a poor molding capacity, insolubility in organic solvents, brittleness, and a limited size in some cases [60]. The disadvantages and limitations of some inorganic piezoelectric materials also include brittleness under mechanical stress [61], the complexity of the manufacturing processes, their less flexible nature, high production costs, and a low Curie temperature [62]. The inherent brittleness of these materials makes them prone to cracking or breaking when subjected to mechanical stress, reducing their durability and reliability in certain applications [63]. Additionally, the complex manufacturing processes required for these materials can contribute to higher production costs and limit their widespread adoption. The less flexible nature of inorganic piezoelectric materials is a drawback in applications that demand conformability to irregular shapes or flexibility in movement [64]. The high cost associated with the production of these materials can hinder their use in cost-sensitive applications, restricting their scalability [64]. Furthermore, some inorganic piezoelectric materials exhibit a low Curie temperature, meaning that they lose their piezoelectric properties at relatively low temperatures, which limits their suitability for high-temperature environments. These factors collectively constitute the challenges and constraints faced by inorganic piezoelectric materials in various practical applications.

**Figure 1:**
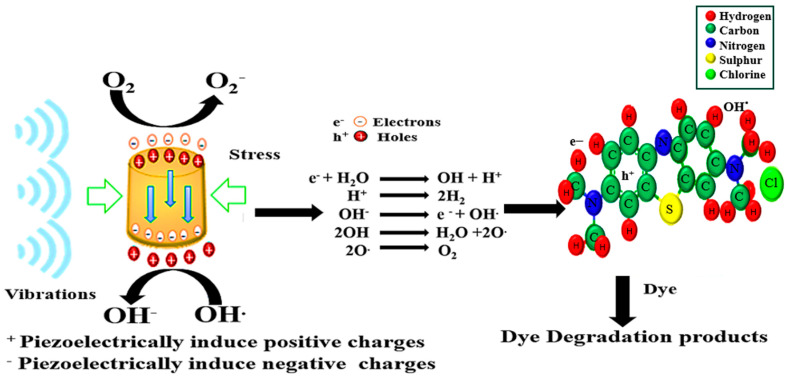
Piezoelectric material under external vibration with separate electron e^−^ and hole h^+^; this separation results in the formation of reactive radicals and causes piezo-catalytic dye degradation.

Figure 1 explains how an electron–hole pair is generated under ultrasonication stress, which creates a reaction with OH and O_2_ and converts them into reactive radicals such as O_2_^−^ and OH^−^, respectively, causing the degradation of organic dyes. If the external stress is removed, the O_2_^−^ and OH^−^ radicals become insufficient for the efficient catalytic degradation of dyes.

### 2.3. Organic Piezoelectric Materials

Piezoelectric materials can be categorized into carbon-based piezoelectric materials and polymer-based piezoelectric materials.

#### 2.3.1. Carbon-Based Piezoelectric Polymers

Carbon-based inorganic 2D materials are very important piezoelectric materials due to their size and shape [65]. Fullerenes, graphene, quantum dots, and nanotubes of carbon may be modified to exhibit piezoelectric properties. Graphene has a monoatomic layer of carbon atoms with flexoelectric and piezoelectric properties [66]. The honeycomb-like structure of graphene shows symmetry, good electric conductivity, strength, and flexibility, but it does not exhibit piezoelectric properties, which can be generated by disturbing the graphene structure. By introducing impurities and defects in the graphene structure, piezoelectric properties can be induced for the production of novel electrochemical devices. DFT studies of graphene [67] doped with K, H, Li, and F atoms were used to calculate the piezoelectric coefficient, which showed high values. This graphene-based piezoelectric material can contribute to the development of nanosized robots, actuators, and motors. The piezoelectric properties of CNTs [68] were also studied and it was proven that a piezoelectric current can be generated by the longitudinal compression or stretching of carbon nanotubes. Strain in carbon nanotubes not only produces a piezoelectric effect but also improves their conductivity and band gap. This investigation proves that carbon nanomaterials can be very helpful modifiers in the production of piezoelectric polymers [69].

**Figure 2:**
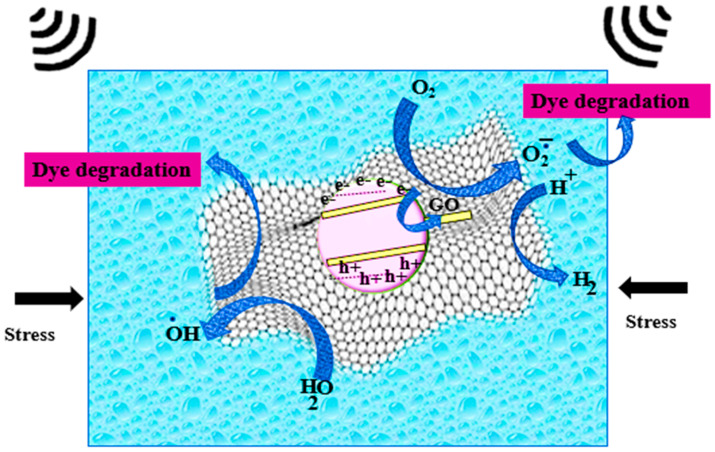
Piezo-catalytic degradation by carbon-based nanocomposites.

Carbon-based nanocomposite materials have improved piezoelectric properties due to the excellent conductivity of carbon-based materials such as CNTs [70] and MWCNTs, which accelerates the charge and transfer of electrons. As shown in Figure 2, when graphene oxide encounters any n-type nanocomposite [71], it forms a heterojunction and piezoelectricity causes electron flow to create a balanced Fermi level. Electron delocalization due to the л-л graphitic network and conductive graphene oxide help to generate reactive oxygen species. These reactive O_2_^−^ and OH^−^ degrade harmful pollutants into harmless products such as CO_2_ and H_2_O.

#### 2.3.2. Polymer-Based Piezoelectric Materials

Polymeric materials can be easily modified and can be obtained in any shape and size [8]. These polymers are flexible, lightweight materials and are resistant to light, temperature, chemicals, environmental conditions, and impurities due to their excellent mechanical properties [72]. There are various piezoelectric polymers but many do not have practical applications yet, although they have potential for the future [73]. These polymers with different morphologies have piezoelectric effects [74]. Nanocomposite polymeric membranes with the addition of different morphologies help to improve the piezoelectric properties of the polymer’s membranes.

## 3. Types of Piezoelectric Polymers

Amorphous and semicrystalline polymers include polytetrafluoroethylene (PTFE) [75], polyethylene terephthalate (PET) [76], polyvinylidene difluoride (PVDF) [66], polyamides [77], polyimides (PI), polyacrylonitrile (PAN), polyolefins, polysulphone (PSF), polycarbonate (PC), and polymethyldisiloxane (PDMS). The practical applications of all these polymers need to be explored in future research. These polymers have a piezoelectric effect due to their extraordinary morphological properties. If these polymers are doped [78] with other conducting inorganic nanomaterials, their electric-charge-carrying ability increases due to the amorphous and semicrystalline nature of the polymer nanocomposite. A piezo-polymeric structure can be obtained by particulate fillers and macromolecular interaction [79].

Stretching extruded films, the use of foaming agents, or multilayered structures help to obtain cellular polymers. A dipole with a proper orientation must be set up during the poling process under a constant electric field. Such dipoles are formed at elevated temperatures and have small piezoelectric coefficients. However, appropriate modification within polymeric material can be conducted to improve their piezoelectric parameters [80]. One of these polymers, polyvinylidene difluoride (PVDF), is a macromolecular compound that has broad applications and it has become the subject of intensive research in recent years [81].

### Polyvinylidene Difluoride (PVDF)

Polyvinylidene difluoride is a semicrystalline polymer with a simple chemical structure [82]. The structure of polyvinylidene difluoride [83] has fluorine atoms on both sides of the hydrocarbon chains. It exists in the most stable nonpolar form with fluorine atoms on both sides of the hydrocarbons; therefore, it does not exhibit a piezoelectric effect. However, the beta phase of PVDF shows a piezoelectric effect, because it has fluorine atoms located on the same side as the chain and forms a permanent dipole [6].

The α-phase to β-phase transformation of PVDF is very important for its piezo-catalytic activity and an aligned β phase can be achieved via the poling of PVDF films [6]. The alignment of the α phase to the β phase causes fluorine and hydrogen atom arrangements so that they are opposite to each other and creates a dipole in the PVDF, which can form a stable heterojunction with a nanocomposite metal oxide and contribute to better electron–hole pair generation.

**Figure 3:**
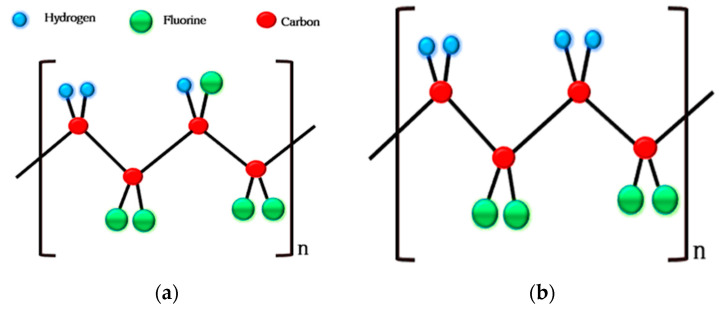
(**a**) α-phase PVDF and (**b**) β-phase PVDF transformation.

**Figure 4:**
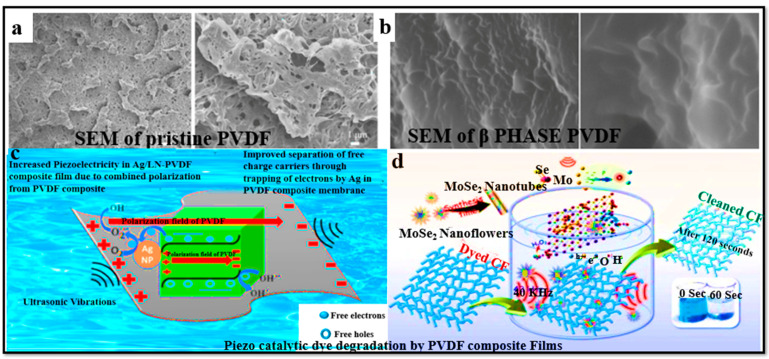
SEM images of (**a**) pristine and (**b**) composite PVDF membranes. (**c**,**d**) Piezo-catalytic degradation of dyes by PVDF composite films.

Figure 3 illustrates that the alpha to beta structure of PVDF under specific conditions makes it very interesting in the field of piezoelectric materials from a technological point of view [84]. The alpha to beta transformation and kinetics can be confirmed by Fourier-transform infrared (FTIR) spectroscopy. The d_33_ coefficient (piezoelectric coefficient) of PVDF = 13 ± 28 Pc/N is comparatively [81] higher than that of other polymers but less than that of ceramic materials. When some suitable inorganic materials are doped with PVDF, they may increase the dipole moment [85]. A modeled β-phase PVDF [86] results in different theoretical calculations of its quantum chemistry, which helps to provide different piezoelectric values. When the dielectric permittivity e^−^ = 5 (which indicates how well a material can be polarized by an electric field), the d_33_ range was found to be 716.6 to 719.2 pC/N; for the dielectric permittivity e = 10, its range was found to be 733.5 to 738.5 Pc/N [87]. The redistribution of molecular orbitals results in the displacement of atomic nuclei and the reorganization of charges under an electric field, which causes a negative piezoelectric effect. The morphological structure of PVDF can be observed at the atomic level by scanning electron microscopy, as shown in Figure 4a,b. Scanning electron microscopy can reach an atomic resolution below 0.05 nm, so it is very helpful in determining PVDF’s morphology and the locations of atoms in PVDF segments [84]. Figure 4 c,d also demonstrate the utilization of piezoelectric catalytic membranes sand materials for dye degradation via the generation of free radicals through piezoelectric activity.

SEM images of pristine PVDF and nanocomposite PVDF are very helpful in the determination of the morphology of polyvinylidene difluoride and the positions of atoms in PVDF. This image is a modified version of the image from an article originally published in the Journal of American Chemical Society and the Journal of Hazardous Materials by Gurpreet Singh et al. and Shrikanta Karmarkar et al.

Transmission electron microscope (TEM) images explain the molecular arrangements of rows of CF_2_ groups at 0.25 nm intervals and a 0.2 nm distance of two fluorine atoms attached to the same carbon atom [88]. These TEM images confirm the structure of PVDF due to the structural correlation with the piezoelectric properties of PVDF and its copolymer. Due to the high chemical stability, thermal resistance, abrasion resistance, photostability, and low density of polyvinylidene difluoride, it is not only used in membranes, pipes, and polymer processing but also in protective coatings, automotive components, and electronics. PVDF and its copolymers can be used as membranes for the exchange of electrons in fuel cells [89]. A theoretical piezoelectric description of PVDF and its modeling concluded that PVDF depends on the temperature and cyclic frequencies, which enable it to be used in sensors and energy harvesting applications. The energy produced by body movements can be transformed into electric energy and used in sensors. Such transducers can be placed in clothes or the human body [90].

The voltage can be calculated by the formula given below:

V = t/Ꜫ d_31_Y ∆L/L (1)

where t = thin film thickness, Ꜫ = absolute permittivity of the material, d_31_ = charge piezoelectric coefficient, Y = Young’s modulus, and ∆L/L = elongation of polymeric film.

Such wearable sensors are very cheap, practical, and non-invasive. The measured pulse can be sent to a PC or smartphone for the analysis of the signals. The use of PVDF-based energy harvesters has proven that they can be used as materials for intelligent road equipment and even to measure the speeds of vehicles [91]. This relies on smart piezoelectric PVDF-based speed bumps, which can act as wireless sensors or current storage batteries. Steel springs in such inverters may store a current under loading conditions and may release it when returned to the original position. The power-generating capacity of such speed bumps is 4 mW cm^−2^ with 0.74% strain at 13 Hz. Such retarding bumps help to achieve the sustainable recovery of energy from car traffic [92]. Gusarov et al. focused on obtaining electricity by heating PVDF-based piezoelectric elements and Ni, Ti, and Cu alloy-based shape memory materials [93]. Table 3 describes the different methods used to manufacture PVDF polymer membranes, along with their properties and applications.

**Figure 5:**
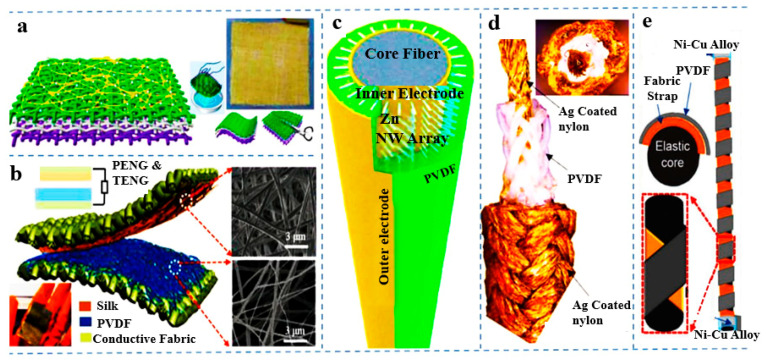
PVDF-based textiles as piezoelectric materials [90]. (**a**) PVDF NFs on top of PET fabric to obtain a highly flexible, tailorable, breathable, and washable fabric, designed by slicing two PET materials in half and placing a conductive fabric between them. (**b**) A piezoelectric and triboelectric hybrid nanogenerator (PTHNG) fabricated entirely of nanofibers by electrospinning PVDF and silk fibroin nanofibers on conductive textiles. (**c**) A piezoelectric nanogenerator with ZnO nanowires and a PVDF polymer as a piezoelectric layer. (**d**) A triaxial braided PVDF yarn harvester made by braiding nylon yarns and coated with a conductive and piezoelectric PVDF melt. (**e**) An elastic core with a piezoelectric polymer PVDF on a helical structural fiber-based PENG. This image is a modified version of an image from an article originally published in the Journal of Advanced Materials by Kai Dong et al.

The piezoelectric effect of PVDF (up to 97% in beta phase) was also studied in textile structures [101]. Figure 5 shows the different PVDF-based textiles that can be used as piezoelectric materials. A textile composed of polyvinylidene fluoride yarns was produced by 2D and 3D weaving [102]. The degree of crystallinity was determined by differential scanning colorimetry (DSC), and the beta-phase content in PVDF was calculated from the FTIR spectra. The formula used in the measurement of the beta-phase content by FTIR is F(β) = Aβ/1.26Aα + Aβ, where Aα and Aβ are the absorption bands at 766 cm^−1^ and 840 cm^−1^. The output voltage during comparison can be determined by dynamic mechanical analysis (DMA). The voltage obtained by 3D weaving was many times greater than that of the 2D weave structure. Polyvinylidene difluoride (PVDF) shows better properties for electroactive materials and has vast applications in the energy sector. There are various methods to enhance the beta phase of PVDF [103].

## 4. β-Phase Induction in PVDF

**Figure 6:**
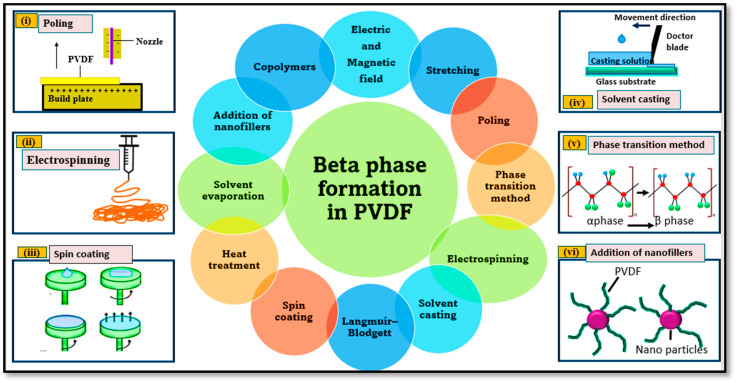
Different methods to enhance electroactive phase of PVDF for better piezocatalytic properties and improved dye degradation. (**i**) Poling. (**ii**) Electrospining. (**iii**) Spin coating. (**iv**) Solvent casting. (**v**) Phase transition method. (**vi**) Addition of nanofillers.

The β-phase content in PVDF is responsible for its piezoelectricity. Several methods can be used to induce the β phase in the PVDF polymer [103], including poling, electrospinning, spin coating, solvent casting, phase transitions, the addition of nanofillers, the application of electric and magnetic fields, stretching, the Langmuir–Blodgett method, heat treatment, solvent evaporation, and copolymers, as illustrated in Figure 6. These approaches aim to enhance the beta phase of PVDF, leading to improved piezo-catalytic properties and dye degradation. A significant enhancement in the β-phase content of PVDF was observed after the addition of fillers in the PVDF polymer matrix, such as ceramics, carbon-based materials, metal oxides, and graphene [6]. Some of these methods to enhance the beta phase of PVDF are stretching [104], electric poling [48], solvent casting, the addition of nucleating fillers, phase transitions, or copolymer development [105].

### 4.1. Stretching

The crystalline transformation of PVDF to generate piezoelectricity can be obtained by thermal annealing with the application of a high voltage via mechanical orientation [106]. Dynamic alignment in the amorphous strands of the polymer can be formed by stretching. This results in uniform crystallite rotation via the application of an electric field. Stretching not only influences the electric but also the mechanical properties of PVDF polymers [107]. The normal α-phase content of PVDF can be converted to the β phase by uniaxial or biaxial stretching. According to previous studies, the temperature also plays an important role in achieving this purpose. A 50 to 140 °C temperature causes effective transformation within the polymer structure [52].

### 4.2. Poling

**Figure 7:**
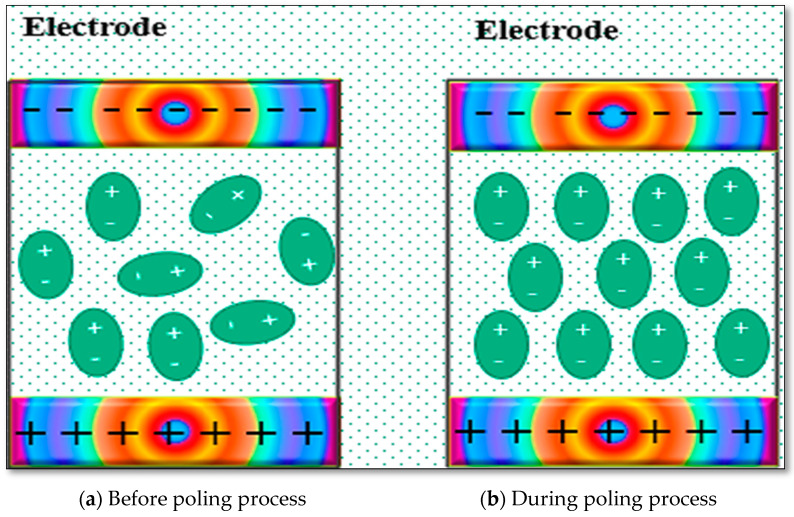
Schematic diagram of PVDF polarization: (**a**) before poling process, (**b**) during poling process.

The piezoelectricity can be enhanced by poling at elevated temperatures, which causes the reorientation of the hydrogen and fluorine atoms of the PVDF structure in the direction of the applied electric field [108]. Strong piezoelectric properties can be achieved by the polarization of polyvinylidene difluoride polymers. Poling helps to arrange the randomly oriented dipole moments in the polymer matrix. As can be observed in Figure 7a, the positive terminals are not aligned in the direction of the applied electric current. However, Figure 7b illustrates the alignment of the terminals in the direction of the applied electric field after the poling process. The best piezoelectricity can be obtained by applying an electric field of up to 200 MV/m, which is difficult to achieve [109]. According to the literature, 160 MV/m is the maximum electric field that is usually set for the electrical poling of PVDF.

The two most commonly used methods for the poling of polymers are the direct contact method and corona method.

**Figure 8:**
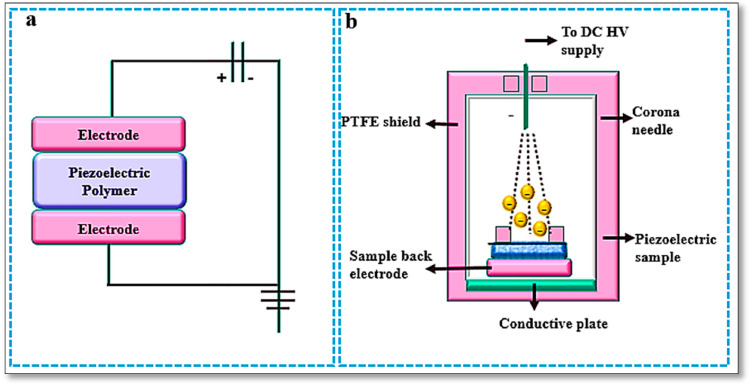
(**a**) Direct poling of polymer. (**b**) Corona poling setup.

(a) Direct contact method: In this method, a high voltage is applied through a conductive electrode across the polymer (Figure 8a). The voltage used in the direct contact method should be a sinusoidal DC or triangular, low-frequency AC waveform. The range of voltages of the applied electric field required across the polymer chain is 5 to 100 ΜV/m. Factors that affect the poling in polymeric materials are the time and strength of the applied electric field, impurities, and the uniformity of the polymer surface [91].

(b) Corona method: Corona poling is a 3D method of poling in which at least one end of the polymeric film is secured with or without an electrode. Corona poling requires the application of a conductive needle at a lower voltage above the piezoelectric polymer. Figure 8b shows a high voltage of 8–20 kV applied by a conductive needle at a much lower DC voltage in a grid above the piezoelectric polymer in dry air. The ionized gas molecules are attracted towards the polymer surface and the number of deposited charges on the polymer surface is controlled by the applied voltage and the position of the grid. Usually, commercial PVDF films are fabricated by this electric poling method [91].

### 4.3. Addition of Fillers

For energy harvesting piezoelectric devices, the addition of fillers in PVDF helps to enhance the piezoelectricity. The effects of different factors and manufacturing techniques also assist in the piezoelectric enhancement of PVDF composites [89]. A simple fabrication process is advantage, allowing different nanofillers and substrates to be combined with PVDF. Improved piezoelectricity is obtained by different hybrid compositions of its structure and the output power from the energy harvesting device.

**Figure 9:**
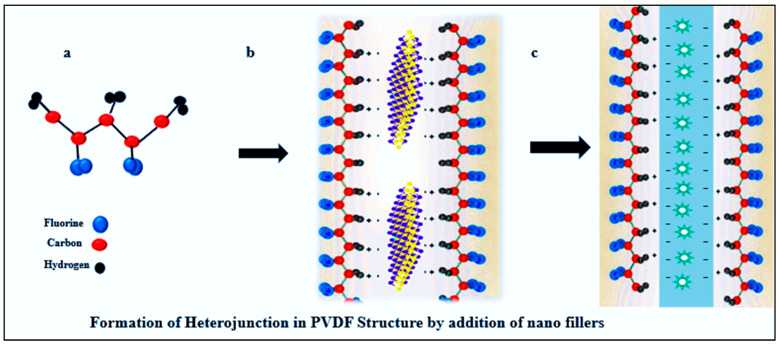
Mechanism of heterojunction formation in PVDF by addition of nanofillers. (**a**) PVDF matrix. (**b**) Nanofillers. (**c**) Formation of the beta phase in PVDF.

As illustrated in Figure 9, when the PVDF matrix (a) is combined with nanofillers (b) such as metal oxides, alignment occurs where the negative side of the metal oxide faces the hydrogen of PVDF. This alignment induces the formation of the beta phase in PVDF (c), facilitating charge transfer and rendering it a more advantageous material for piezoelectric applications. The addition of nanofillers also reduces the recombination of electrons and holes to combine with oxygen and H_2_O and helps to produce reactive radicals for the effective degradation of organic effluents. PVDF films can be synthesized by the simple solvent casting method [110], in which the polymer mixture is stirred at a 60 °C temperature. The polar solvents that are used to dissolve PVDF are dimethyl formamide (DMF), N-methyl pyrrolidine (NMP), dimethyl sulfoxide (DMSO), and dimethylacetamide (DMAc). An improved beta-phase orientation and uniform area of the film can be developed by the spin coating technique, which involves the high mechanical stretching of the film [111]. Spin rotation, the annealing temperature, and the solution concentration are the parameters that contribute to β phase formation in PVDF films. Quenching in water at room temperature and spin coating at a low temperature are factors that have led to improved piezoelectric effects. One of the most popular methods used for PVDF fiber casting is electrospinning, and many researchers are working on the synthesis of PVDF fibers by electrospinning. A better piezoelectric coefficient and better dielectric properties can also be obtained in electrospun PVDF fibers [105].

Table 4 illustrates the different methods to enhance the beta phase of PVDF for improved piezoelectricity. The most energy-efficient method among those discussed above is the incorporation of nanofillers into PVDF. These nanocomposites exhibit piezo-catalytic activity without relying on an electric current or any external sources of energy to initiate the process. This strategy not only underscores the shortcomings of alternative methods but also provides insights into the mechanisms that enhance the piezoelectric performance.

## 5. Energy-Efficient β-Phase Enhancement in PVDF

**Figure 10:**
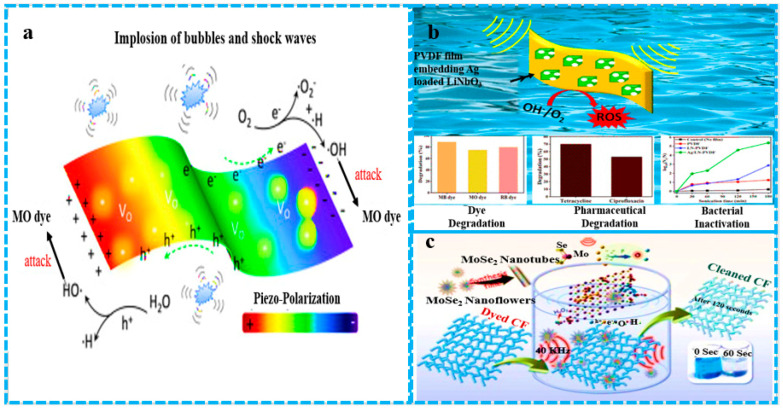
Piezo-degradation of pollutants by generation of reactive oxygen species. This image is a modified version of various images from articles published in the Journal of Cleaner Production by Qing Nie et al., the Journal of the American Chemical Society by Gurpreet Singh et al., and the Journal of Hazardous Materials by Srikanta Karmaker et al.

PVDF conversion from the alpha to beta phase is usually obtained by stretching [117] or poling [118] under a high electric field. The addition of nanofillers is another effective method that helps to induce the beta phase in PVDF. The solution casting method is very useful for beta crystal formation in the PVDF matrix with addition of a special filler that helps to improve the piezoelectric ability of PVDF membranes. Carbon-based nanofillers, clay particles, or modified nanotubes also help to induce the beta-phase transformation in PVDF nanocomposites [119]. Many carbonyl groups containing fillers cause the homogeneous dispersion of the PVDF matrix due to the interaction of fluorine in PVDF and carbonyl (C=O) on the filler surface. These interactions not only induce the beta phase but also increase the thermal, mechanical, and electric properties of the nanocomposites. In different graphene oxide-based nanocomposites, single layers of GO sheets contain oxygen-containing functional groups, such as hydroxyl, epoxy, and carboxyl groups, which give hydrophilic characteristics to graphene oxide [120]. PVDF, when mixed with graphene oxide nanosheets in a DMF solution by the solution casting method, showed high compatibility and good dispersion in the PVDF polymer matrix. This piezoelectric polymorph combination produces a β polymorph of PVDF with increased piezoelectric properties [119]. Piezoelectric materials, when subjected to mechanical stress, can produce an electric charge. This electric charge produced in piezoelectric materials due to mechanical stress generates free charges on the ambient interface, where these charges react with oxygen to generate reactive radicals such as OH^•^, O^•−^, HO_2_, and H_2_O_2_ via the local micro-electrolysis of water. These reactive oxygen species generated by piezoelectric materials can be efficiently used to degrade toxic organic dyes in water, especially from the pharmaceutical, chemical, and textile industries [23]. Figure 10 shows the piezo-degradation of pollutants by the generation of reactive oxygen species. Piezoelectric polarization induces the generation of free radicals, highlighting the potential of incorporating fillers into PVDF-based catalytic membranes for combined filtration and degradation purposes. Figure 10a depicts the efficient degradation of MO dye facilitated by the piezoelectric activity of thin nanocomposites. Figure 10b illustrates the degradation of pharmaceuticals and bacteria by a nanocomposite (LiNbO_3_/Ag) PVDF membrane utilizing the piezoelectric effect induced by ultrasonication. Additionally, Figure 10c highlights the rapid degradation of dyes within 60–120 s under low-frequency ultrasonication, achieving approximately 86% degradation efficiency for both rhodamine B (RhB) and methylene blue (MB) via piezo-catalysis using FMNs.

Piezoelectric properties can be improved for better piezo-catalytic activity by adopting different synthetic schemes and nanocomposite fillers. These improved properties can be applied for different applications and help to achieve the benefits of energy-efficient piezo-catalytic materials with net-zero emissions. Table 5 illustrates the different fabrication methods and materials used to improve the performance of PVDF-based composites in piezo-catalytic applications.

The piezoelectric mechanism can be improved by photocatalytic metal oxide ions and piezoelectric PVDF polymer composites. The electric field produced in hydrophobic PVDF films helps to improve the separation of the photoinduced carriers of metal oxide nanostructures [125]. The piezoelectric field generated in PVDF by mechanical action can improve the catalytic efficiency of photogenerated carriers. Shuwei Han et al. described the piezotronic effect in a Sn_3_O_4_/PVDF hybrid photocatalyst driven by water flow, which could be used for water purification applications [126]. The output performance of the generator depends on the processing conditions of the nanofillers in the PVDF matrix, which can result in the improved development of the beta phase. The beta (β) phase in PVDF membranes shows superior membrane fouling resistance compared to the α phase of PVDF [127].

## 6. Energy-Efficient Piezo-Catalytic Membranes

Piezoelectric membranes use vibrational energy as the external source, such as ultrasonic waves, which helps significantly in the reduction of membrane fouling [127]. Piezoelectric membranes are flexible composite films that help in the filtration of water, and the piezo potential of these films helps in the transfer of charge carriers. When nanocomposites are used in these piezoelectric membranes, they also help in the degradation and elimination of water contaminants. Piezo-catalytic membranes boost superoxide radical formation synergistically with photocatalysis, which can cause improved charge separation, and they show great potential in the control or elimination of water pollutants [128]. The piezo-electric properties of membranes can be enhanced by various factors, such as poling [129,130,131], the temperature [132], and the nature of the solvent.

### 6.1. PVDF-Based Piezo-Catalytic Membranes for Water Purification

Piezoelectric membranes are fabricated from semicrystalline polyvinylidene fluoride (PVDF), which is an ideal material due to its piezoelectric properties [133]. The chemical and mechanical resistance of PVDF make it an attractive polymer for the fabrication of membranes. The mechanical, thermal, and chemical properties of PVDF, with favorable processing parameters, are helpful in the performance of PVDF membranes for wastewater treatment. Self-cleaning PVDF membranes can be fabricated with 0D, 1D, and 2D hybrid materials for water purification [134]. The piezoelectric properties of polyvinylidene difluoride, including all known properties of the PVDF polymer (α, β, γ, δ), are demonstrated by the all-trans (β) phase of PVDF [30]. The β phase of PVDF can be fabricated by methods such as crystallization under high pressure [135], stretching [136], poling [137], the blending of polymers [138], electric poling [137], and the addition of various additives [139]. Table 6 shows the various methods used to fabricate piezo-catalytic β-phase PVDF membranes for water treatment.

### 6.2. Factors Affecting Piezo-Catalytic Membranes

There are some factors that may have a strong impact on piezo-catalysis. These factors include the nature of the mechanical field, its frequency, and the redox potential of the reactions. Piezo-catalytic reactions are driven by piezoelectric forces, unlike electric and photocatalytic stimuli. The piezo-catalyst’s mechanisms include the acoustic field in which redox reactions take place due to potential differences [145]. Dissolved oxygen reacts with the negative charge e^−^ to produce O_2_^−^. Superoxide radicals and hydroxide radicals form a positive charge h^+^ and hydroxide ions. Another phenomenon responsible for piezo-catalytic activity is localized superficial polarization. This localized polarization in micro- and nanostructures leads to an electric potential difference in the piezo-catalyst structure, which causes piezo-catalysis [146]. However, further research is required on a nano and micro scale for a better explanation of piezo-catalysis. The valence and conduction bands of piezoelectric catalysts favor redox reactions under ultrasound and sonication. Sonochemistry uses ultrasound of high and low frequency for the degradation of organic pollutants. Sonochemical treatments have been very successful in the degradation of organic pollutants. Sonochemistry [147] is strongly related to piezo-catalysis, and it should not be neglected while performing experiments. The effect of the presence or absence of ultrasound and catalyst radical scavengers on the piezo-catalytic surface can be tested for a better understanding of piezo-catalysis. The various factors that may affect piezo-catalysis include the size, shape, and geometry of the material; the piezoelectric coefficient d_33_; poling; the ultrasonic power; the acoustic field; and the temperature [145]. Table 7 elaborates the different factors that regulate the mechanism of piezo-catalysis, influence the phenomenon, and play a crucial role in the degradation of catalytic dyes.

By effectively controlling the above factors, we may achieve a better piezo-catalytic response for dye degradation. Dye degradation can be achieved in the desired time by effectively controlling the above parameters [26]. Dye degradation in a limited period can also be obtained by exploring the effects of all factors.

### 6.3. Mechanism of Dye Degradation by Piezo-Catalysis Membranes

Piezo-catalysis is an advanced oxidation process in which reactive oxygen species such as superoxide (^•^O_2_^−^), hydroxyl radicals (^•^OH), and singlet oxygen are generated by high-energy ultrasound waves or low-frequency vibrations [150,151,152]. During piezo-catalysis, electron and hole pairs are generated and undergo piezoelectric redox reactions. During water purification, these ROS undergo oxidation and reduction reactions with dissolved pollutants. The generation of reactive oxygen species by piezo-catalytic materials depends on the piezoelectric coefficient, d_33_, and is directly prepositional to d_s_ [153,154]. Some classes of dielectric materials exhibit piezoelectric properties when mechanical energy is applied. Piezo-polymeric materials such as polyvinylidene fluoride (PVDF) with nanocomposites achieve an increased piezo-active phase. Stress induces piezo potentials in such membranes, due to which free electrons and holes produce separate charges effectively in opposite directions [155]. Periodic ultrasonic irradiation helps to deform doped nanofibers or conducting polymeric materials in the matrices of PVDF membranes [156].

Charge separation in PVDF and doped materials induces an electric field, which increases the catalytic effect. Nanocomposites inside the membrane matrix may generate heterojunctions and reduce charge recombination. Thus, the CB electrons can capture the absorbed O_2_ to produce O_2_^−^. Electrons can react with the absorbed O_2_ and H_2_O_2_, which can produce OH radicals, which help to degrade organic compounds such as dyes into carbon dioxide and water [157]. The beta phase of PVDF helps in the degradation of dyes, which can be monitored by UV–visible spectroscopy. During piezo-catalytic degradation, ultrasonication helps to degrade dyes, even in the dark, without any influence of photo-catalysis [23]. Pristine PVDF membranes do not show dye degradation, even under prolonged sonication. However, nanocomposite PVDF films show excellent piezo-catalytic dye degradation. Nanoparticles applied in PVDF membranes help in self-poling piezo-catalytic membranes [9,23]. When these piezoelectric membranes are subjected to ultrasonic vibration in dye-containing water samples, the polarization of the composite membrane generates electron–hole pairs on the surface. Continuous ultrasonication by rapid vibration helps in the generation and accumulation of electron–hole pair on membrane surfaces and splits water to form reactive oxygen species such as OH and ^•^O^−^ radicals [158]. This mechanical stimulus generation of OH^•^ radicals in the dye solution can be detected by electron spin resonance (ESR) [148] by introducing different trapping agents at regular intervals, such as terephthalic acid upon reaction with OH^•^ Radicals to form fluorescent hydroxy terephthalic acid, which can be detected by qualitative means [159]. 

**Figure 11:**
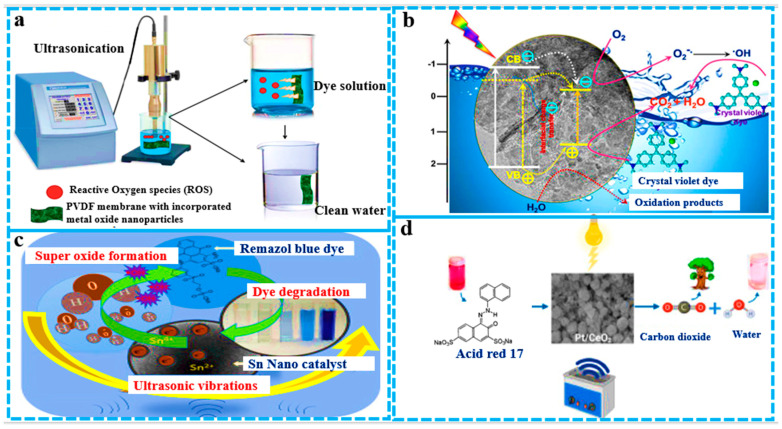
Schematic diagrams of generation of reactive oxygen species (ROS) by ultrasonication and catalytic degradation of dye. This image is modified from an article related to piezo-catalysis from the Journal of Nano Energy by Biswajoy Baghchi et al., a Journal of Chemistry article by Rao Akshatha et al., a Journal of Materials Chemistry and Physics article by Sakthivel Jayaraman et al., and a Journal of Molecular Structure article written by Muhammad Farooq Khan.

Figure 11 illustrates the primary mechanism of organic effluent degradation, wherein catalytic materials generate reactive oxygen species (ROS) to facilitate pollutant degradation in water, resulting in the formation of less harmful by-products. Figure 11a Ultrasound in dye water on a PVDF/MoS_2_ membrane induces electron–hole pairs, enhancing ROS production for rapid dye degradation. Figure 11b The MoS_2_/wurtzite ZnS catalyst demonstrates exceptional performance in the photocatalytic degradation of crystal violet dye, primarily driven by superoxide formation, highlighting its effectiveness for environmental remediation and hydrogen production. Figure 11c Ultrasound-assisted degradation with Sn nanoparticles achieves 85% efficiency in 10 minutes through rapid hydroxyl radical production and electron interaction. Figure 11d The efficient degradation of acid red 17 by a Pt/CeO_2_ nanocomposite under 40 kHz ultrasonic waves and visible light, facilitated by heightened OH^•^ free radical production.

Recent advancements in piezo-catalytic membranes also demonstrate the capability to reuse these membranes for many cycles with more than 90% dye degradation efficiency. Thus, these PVDF membranes can provide the reliable and safe production of reactive oxygen species (OH, O^−^), which can be applied for the degradation of organic and inorganic pollutants from wastewater reservoirs, sewer systems, and drainage systems, where photocatalytic applications are difficult to implement. The independence of light and electricity makes the piezoelectric phenomenon a unique energy conservation phenomenon.

## 7. Piezoelectric Phenomena as Net-Zero Emission Source

Piezo-catalysis is a green catalytic process due to its independence of light and electricity [11]. The use of mechanical energy by tides, winds, and human activity causes deformation in non-centrosymmetric materials and generates positive and negative charges. The redox potential of the reaction and frequency are important factors that affect the piezo-catalytic phenomenon. The piezo-catalytic phenomenon is considered as a new, advanced oxidation technique in which high-frequency ultrasound waves and low-frequency vibrations set up a built-in electric field, which separates electron–hole pairs [148]. The superoxide radical ^•^O^−2^ is generated by the reaction of electrons with dissolved oxygen in water, while the hydroxide ion OH is formed by the positive charge (hole h^+^) and hydroxide ions. These free radicals degrade organic compounds into CO_2_ and H_2_O, which can be recycled as fuel. Different piezoelectric materials are being investigated for CO_2_ reduction, hydrogen evolution, and the degradation of organic pollutants [160]. The CO_2_, H_2_, and H_2_O yielded as a result of dye decomposition are not harmful pollutants and can be reused for the production of fuel [161]. CO_2_ reduction and H_2_ evolution from water can be intermittently used to create energy resources. Mechanical vibration serves as an energy-efficient method without the need for an external energy input, leading to the degradation of dyes via free radicals. The efficient recapture and reuse of these degradation by-products can significantly contribute to the concept of net-zero emissions.

**Figure 12:**
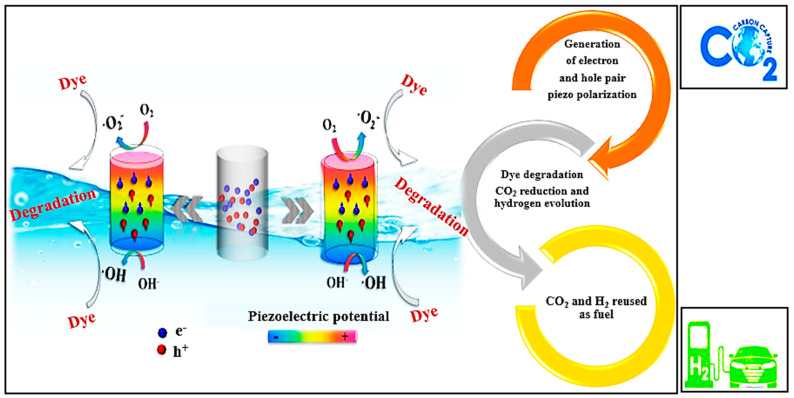
Piezo-atalysis for degradation of organic dyes and production of CO_2_ and H_2_ as fuel. This image is modified from an article by Bian yang et al., from the Journal of Material Chemistry.

Figure 12 illustrates the mechanism of piezo-catalysis for the degradation of organic dyes. Piezo-catalytic degradation can help in hydrogen and CO_2_ production by dye degradation, which is the best example of net-zero emissions. Water purification by mechanical energy sources not only helps to degrade organic pollutants but also yields carbon dioxide and water. These by-products may be used as fuel after proper capturing. Scavenger tests are usually performed for the confirmation of the generated free radicals by using various trapping reagents. Table 8 provides an overview of the reported works illustrating how the addition of PVDF nanocomposites leads to the generation of free radicals, as confirmed by scavenger tests, and results in the degradation of dyes into simpler by-products. This progress also aligns with the objective of net-zero emissions.

## 8. Conclusions and Future Scope

This review article explores recent energy-efficient techniques employed in the removal of various toxic contaminants present in textile wastewater. Given the global issue associated with energy, the recycling of energy resources through the implementation of energy-efficient methods emerges as a promising solution. Recent reviews suggest that novel piezoelectric polymers, biopolymers, and their nanocomposites hold potential for water purification via the catalytic degradation of organic pollutants. Piezo-catalytic dye degradation represents an energy-conserving approach to the breakdown of organic pollutants. Among various piezoelectric polymers, polyvinylidene difluoride (PVDF) stands out due to its exceptional mechanical and piezoelectric properties.

Self-poled nanocomposite piezoelectric membranes composed of PVDF have shown great promise in terms of energy efficiency. These membranes possess the ability to generate reactive oxygen species when immersed in an aqueous solution, thanks to the induction of β crystals in the PVDF matrix, which leads to spontaneous orientation and dipole formation. They offer robustness and reusability, making them ideal for purifying large volumes of dye-contaminated water. However, despite their potential, the sensitivity of these piezo-catalytic membranes to mechanical forces presents a significant challenge that needs to be addressed for improved energy efficiency. Therefore, further research is required to understand and overcome this limitation, in order to fully harness the benefits of these membranes in large-scale water purification applications.

Achieving the desired ultrasonicated vibrations necessary for piezo-catalytic membranes is challenging due to their limited occurrence in natural environments. Moreover, these flexible polymeric membranes can undergo deformation when subjected to stress. However, these polymers exhibit lower stability and a slower piezoelectric response and are less cost-effective compared to inorganic piezoelectric materials.

To address these limitations, several key areas require focus.

(1) The development of new, stable, and cost-effective piezoelectric materials with enhanced sensitivity is essential. Exploring alternative mechanical energy resources can facilitate the creation of attractive, energy-efficient polymeric materials with specific morphologies and designs.
(2) A comprehensive understanding of the underlying mechanisms through theoretical calculations is needed, including charge transfer, the electronic structure, and the kinetics of piezo-catalytic reactions under light and stress influences. Computational and experimental studies are necessary for the quantitative analysis of the relationship between the piezo potential, electronic energy levels, and intricate material interactions. Such knowledge can guide the discovery of highly efficient multifield catalytic systems by elucidating the energy shifts in metals and p-/n-type semiconductors.
(3) Expanding the applications of piezo-catalysts beyond water splitting and pollutant degradation is crucial; there is a need to explore applications such as CO_2_ reduction, nitrogen fixation, medical treatments, and selective organic synthesis, which will contribute to practical solutions for energy and environmental challenges. Developing stable and recoverable piezoelectric materials holds promise in addressing these issues.
(4) Advancements in piezo-catalysis can pave the way for sustainable approaches to wastewater treatment. Future researchers can explore smart and advanced fabrication approaches for piezoelectric membranes, enabling the capture and reuse of degraded CO_2_ and H_2_. This review not only emphasizes the utilization of natural energy sources to tackle global challenges but also highlights the potential to reuse by-products as an energy source.
(5) Achieving net-zero emissions through highly efficient catalytic activity driven by multiple irradiation sources and natural activities would be a significant step forward in pollutant degradation. Further advancements in membrane design and application hold immense potential but also present open challenges and expectations for piezo-catalytic membrane technology. Continued research and innovation in this field are necessary to unlock its full capabilities.

## Figures and Tables

**Table 1:** PVDF-based polymeric nanocomposite membranes for dye degradation.

Polymer Nanocomposite Membrane Fabrication	Method of Fabrication	Shape	Dye Degradation	Ref.
PVDF/GO	Electrospinning/modified Hummer’s method	Nanofibrous membranes	99%	[22]
PVDF/MoS_2_	Solution processing/hydrothermal process	Flat sheet with uniform distribution of nanoparticles	90%	[23]
PVDF/ZnO	Electrospinning	Nanofibrous membrane	99%	[24]
PVDF/ZnSO_3_, Co_3_O_4_	Simple blending hot molding/hydrothermal	Simple blending hot molding	99%	[5]
PVDF/PVA blended with NiO NPs	Casting technique	Nanocomposite membrane	95.4%	[25]
PVDF/barium titanate (BaTiO_3_, BTO)–polydimethylsiloxane (PDMS) composite	Electrospinning method	Nanofibrous membrane	94%	[26]
PVDF/BiVO_4_–GO	Ultrasonic method/hydrothermal method	Resembles a human embryo embedded inside an amniotic sac	99%	[27]
PVDF/Ag_2_CO_3_	Solution processing/co-precipitation method	Flat sheet with uniform distribution of nanoparticles	80%	[28]
Ag@LiNbO_3_/PVDF	Solvent casting method	Flat sheet	90%	[29]
Ba_0.85_Ca_0.15_Zr_0.1_Ti_0.9_O_3_ (BCZTO) ferroelectric ceramic particles/PVDF	Polymerization method	Flat sheet	91%	[30]

**Table 2:** Inorganic piezo-catalysts, their applications, and the conditions required to degrade organic pollutants.

Inorganic Piezoelectric Material	Fabrication Method	Shape	Application	Condition	Performance	Ref.
Barium titanate (BaTiO_3_) NPs	Hydrothermal process	Tetragonal	Degradation of organic pollutants	Ultrasound (110 W, 40 kHz)	67%	[52]
Molybdenum disulfide (MoS_2_) nanoflowers (NFs)/carbon fibers	Hydrothermal process	Nanoflower/fibers	Degradation of organic pollutants	Ultrasound (250 W, 40 kHz)	90%	[53]
2-Methylimidazole zinc salt (ZIF-8) nanoparticles	Liquid phase method	Nano-diamonds	Dye degradation of rhodamine	Vibrations	94.5%	[54]
Flower-like to tube-like microstructures of molybdenum disulfide (MoS_2_)	Solvothermal reaction	230 nm nanoflower	Dye degradation	Ultrasonic vibrations	86%	[55]
Molybdenum disulfide (MoS_2_)/graphdiyne	Ball milling	Nanosheets	Degradation of tetracycline	Piezo-catalysis	87%	[53]
Titanium dioxide (TiO_2_) nanoparticles	Hydrothermal method	Granular-shaped nanoparticles	Degradation of different dyes	Magnetic stirring	99.7%	[56]
Zinc oxide (ZnO) nanoparticles	Facile green solid state chemistry method at room temperature	Nanoparticles, nanorods	Degradation of methylene blue	Ultrasonic vibrations	99%	[57]
Few layers of tungsten sulfide/molybdenum disulfide/tungsten diselenide (WS/MoS_2_/WSe_2_)	Solvothermal method	Nanosheets	Degradation of tetracycline and rhodamine B	Ultrasonic vibrations	90%	[58]
Orthorhombic zinc stannate (ZnSnO_3_) nanoparticles	Colloidal dispersion method	4–5 nm nanoparticles	Dye degradation	Ultrasonic vibrations	100%	[59]

**Table 3:** Different methods used to fabricate PVDF polymer membranes, along with their properties and applications.

Fabrication Method of PVDF Polymer	Physical Properties	Chemical Properties	Thermal Properties	Mechanical Properties	Applications	Ref.
Phase inversion	Colorless	Resistant to organic solvents	Melting point 169 °C	Tensile strength at 23 °C, 35–55 MPa, ASTM D-638 [94]	Sensors and actuators	[95]
						[7]
Use of inorganic particles with polymer solution	1.78 g/cm^3^ density	Resistant to alcohols	Deflection temperature (261 psi) 114–118 °C	Elongation at 23 °C, 25–500 %, ASTM D-638	Spin valve devices	[7,95]
Phase separation using supercritical CO_2_ as non-solvent	Melting point, 177 °C, ASTM D-3418 [96]	Resistant to acids and bases	Oxygen index 43%	Young’s modulus at 23 °C, 1340–2000 MPa, ASTM D-638	Magnetoelectric materials	[7,95]
Electrospinning	Glass transition temperature −35 °C	Resistant to oils and fats	Maximum service temperature 149 °C	Thermal expansion coefficient, ASTM D-696 [97] ~10–4	Energy harvesting applications	[95]
Method of fabrication depends on application	Relative density, 1.76–1.8 g/cm, (solid) ASTM D-792 [98]	Resistant to milk, glucose, vinegar, olive oil	Thermal stability 0.17–0.19 W/m^−K^	Dielectric strength, 260–950 kV/mm, ASTM D-149 [99]	Tissue engineering	[7,95]
-	Heat deflection temperature (0.5 MPa) 148 °C	-	Flammability UL-94 V-O °C	Dissipation factor, 0.0163–0.019 (1 kHz), ASTM D-150 [100]	Membrane technology	[7,95]

**Table 4:** Effect of different methods on PVDF beta-phase enhancement.

Method of Beta-Phase Enhancement	Mechanism Involved in Improving Piezoelectricity	Limitations of the Method	Ref
Stretching	Effective dipole moment alignment	Decrease in crystallinity	[112]
Poling	Induces stress due to electric field	Electric breakdown	[109]
Addition of nanofillers	Increases crystallinity and uniform distribution Less electricity required for poling	Filler aggregation Failure in poling process	[113]
Heat treatment	Realignment of molecular chains by heat treatment	Crystallinity degree increases Expansion of amorphous regions, which causes defect formation	[114] [115]
Filler alignment	Crystallinity degree increased	Suppression of crystallinity process at higher magnetic fields	[116]

**Table 5:** Effects of different methods and materials to enhance piezoelectric behavior of PVDF-based composites.

PVDF Composite Processing Method	Materials/Fillers	Improved Properties	Applications	Ref
Solution casting method	MoS_2_ nanoflowers	Self-poled piezoelectricity	90% dye degradation	[23]
Polyaniline (PANI) electrochemical/zinc ferrite nanorods/hydrothermal method–drop casting technique to prepare a ternary PVDF composite (80 mm)	PANI helps to stabilize the output voltage. It works as a dispersing agent for nanofillers and improves the homogeneity of the filler distribution in zinc ferrite nanorods to improve the mechanical and piezoelectric properties	Output piezo voltage of 4.2 V and enhanced high-power density of 3.56 mw/mm^3^	To charge capacitors, self-powered devices, and sensors	[121]
Simple hot blending technique	PVDF/zinc stannate/cobalt oxide composite	Rhodamine B (RhB) and methylene blue (MB) degradation	100% dye degradation efficiency in 20 mints	[5]
Electrospinning	Inorganic perovskite quantum dots (IPQDs; CsPb_0.25_Zn_0.75_I_3_) with eco-friendly cellulose nanocrystal (CNC) ligands in polyvinylidene fluoride (PVDF)	Pyro-catalytic RhB dye degradation	91% RhB dye decay	[122]
A facile approach to produce plasmonic modulated PVDF/MoS_2_ cavity/Au heterostructure via hydrothermal method	PVDF/MoS_2_ cavity/Au heterostructure	Water purification	99.9% MB degradation within 45 min	[123]
Solvent casting method	Polyvinylidene difluoride with LiNbO_3_ ceramics decorated with silver nanoparticles (AgNPs)	Cationic and anionic dye degradation, pharmaceutical pollutant degradation	99% MB dye degradation 80% RB dye degradation 75% MO dye degradation	[29]
Solution casting method	Ferroelectric Ba_0_._85_Ca_0_._15_Zr_0_._1_Ti_0_._9_O_3_ (BCZTO) ceramic particles were immobilized in a polymer matrix of polyvinylidene difluoride (PVDF)	Methylene blue, rhodamine B, and methyl orange	∼91% MB degradation, ∼86% rhodamine B degradation, and 90% methyl orange degradation 180 min, sonication	[124]

**Table 6:** Methods to fabricate piezo-catalytic β-phase PVDF membranes for water treatment.

Β-Phase PVDF Piezo-Catalytic Membrane	Method of Fabrication	Application	Ref
PVDF/MoS_2_ nanosheets	Electrospinning	Oxytetracycline degradation in water	[9]
PVDF with different morphologies	Non-solvent-induced phase separation method	Improved piezoelectric response	[127]
PVDF composite film embedding LiNbO_3_ ceramics decorated with silver nanoparticles (Ag NPs)	Solvent casting method	Dye/pharmaceutical degradation and bacterial disinfection	[23]
Fe_2_O_3_/PVDF-HFP porous film	Fenton degradation mechanism	Self-powered environment cleaning	[31]
PVDF/MoS_2_ cavity/Au heterostructure	Hydrothermal method	Wastewater treatment	[123]
Glass fiber (FG)-assisted polyvinylidene fluoride (PVDF) hybrid membrane	Solution coating method	Tetracycline degradation and oil–water separation in wastewater	[140]
Bi-piezoelectric ZnO nanorods (NR)/PVDF–HFP spongy film	Phase inversion method	Dye degradation, can be recycled	[141]
Polyvinylidene fluoride (PVDF) nanofiber films containing a small concentration of sodium dodecyl sulfate (SDS)	Electrospinning	Supercapacitor applications	[142]
t-BaTiO_3_/Ag/β-PVDF composite material	Use of compounding powder and polymer	Dye degradation	[143]
Reusable polyvinylidene fluoride (PVDF)–[0.67BiFeO_3_–0.33BaTiO_3_] (BF33BT) composite	Sol–gel and solvent casting method	Dye degradation	[144]

**Table 7:** Roles of various processes in piezo-catalysis and dye degradation.

Factor	Mechanism	Effect on Piezo-Catalysis	Role in Dye Degradation	Ref
Absorption–desorption equilibrium	Attained by dye solution in dark without sonication	Proves effect of vibration on sample	No notable changes in the concentration of the dye without ultrasonication	[5]
Poling	Poling of material causes better charge separation	Poling of piezoelectric material causes better charge separation due to electric field generation	In undoped PVDF film, the dyes remained unchanged even after prolonged ultrasonication, indicating the piezoelectric effect in MoS_2_–PVDF film catalytic process	[146]
Ultrasonic power	Piezo-catalyst excitation	Ultrasonication power has some effects on piezo-catalysis	Calorimetrically calibrated ultrasonic system should be used to determine the ‘real’ acoustic power	[148]
Temperature	Ultrasonication causes gradual increase in temperature	Rise in temperature may affect piezo-catalytic process	Dye degradation affected by change in temperature	[149]
Acoustic field	Acoustic field set up by ultrasonication plays role in piezo-catalysis	Controls reproducibility of the acoustic field while using sonication to excite piezo-catalysts	Better control and understanding of the acoustic field can enable its modulation to enhance piezo-catalytic activity	[145]
Heterojunction	Mechanical deformation helps in transfer of charges	Linear voltage changes across the material	Free charges create piezo potential for dye degradation	[146]

**Table 8:** PVDF nanocomposite membranes used for degradation of dyes to produce CO_2_ and H_2_O.

PVDF Composite	Dye Used	Radical Generated	Scavenger Test	Degradation Product	Net Zero	Ref
MoS_2_–PVDF	Acridine orange (AO), Eosin Y (EO), ethidium bromide (ET), and rhodamine B (RHO) (Lobachemie)	^•^OH hydroxyl radical	^•^OH trapping with terephthalic acid, which forms fluorescent hydroxy terephthalic acid upon reaction with ^•^OH radical	Carbon dioxide and water (CO_2_ + H_2_O)	Reuse of carbon dioxide and water (CO_2_ + H_2_O) as fuel	[18]
PVDF/ZnSn_3_ nanocube/Co_3_O_4_ nanoparticle	RhB degradation	(OH) hydroxyl radical (O^−2^) superoxide radical	Tert-butyl alcohol (TBA), benzoquinone (BQ), and disodium ethylenediaminetetraacetate dehydrate (EDTA)	Carbon dioxide and water (CO_2_ + H_2_O)		[5]
ZnO nanowire/PVDF nanofiber	RhB degradation	(OH) hydroxyl radical (O^−2^) superoxide radical	Isopropanol (IPA), disodium ethylenediamine tetraacetate (EDTA), and benzoquinone (BQ)	Carbon dioxide and water CO_2_ + H_2_O	Reuse of CO_2_ + H_2_O as fuel	[24]
Bi-piezoelectric integration of ZnO nanorods and PVDF	RhB degradation	(OH) hydroxyl radical (O^−2^) superoxide radical	Use of scavengers	Carbon dioxide and water (CO_2_ + H_2_O)	Reuse of CO_2_ + H_2_O as fuel	[162]
BiVO_4_-GO-PVDF nanocomposite	Methylene blue (MB), rhodamine B (RhB), and safranin O (SO)	(OH) hydroxyl radical (O^−2^) superoxide radical	Use of scavengers	Carbon dioxide and water (CO_2_ + H_2_O)	Reuse of CO_2_ + H_2_O as fuel	[27]

## Data Availability

Not applicable.
